# Effect of soft tissue injury and ulnar angulation on radial head instability in a Bado type I Monteggia fracture model

**DOI:** 10.1097/MD.0000000000017728

**Published:** 2019-11-01

**Authors:** Naoki Hayami, Shohei Omokawa, Akio Iida, Tsutomu Kira, Hisao Moritomo, Pasuk Mahakkanukrauh, Jirachart Kraisarin, Takamasa Shimizu, Kenji Kawamura, Yasuhito Tanaka

**Affiliations:** a Department of Orthopedic Surgery; b Department of Hand Surgery, Nara Medical University, Kashihara, Nara; c Department of Physiotherapy, Osaka Yukioka College of Health Science, Ibaraki, Osaka, Japan; d Excellence in Osteology Research and Training Center (ORTC); e Department of Anatomy; f Department of Orthopedic Surgery, Faculty of Medicine, Chiang Mai University, Chiang Mai, Thailand.

**Keywords:** Monteggia fracture, radial head instability, soft tissue injury, ulnar angulation

## Abstract

The effects of soft tissue damage and ulnar angulation deformity on radial head instability in Monteggia fractures are unclear. We tested the hypothesis that radial head instability correlates with the magnitude of ulnar angular deformity and the degree of proximal forearm soft tissue injury in Bado type I Monteggia fractures.

We performed a biomechanical study in 6 fresh-frozen cadaveric upper extremities. Monteggia fractures were simulated by anterior ulnar angulation osteotomy and sequential sectioning of ligamentous structures. We measured radial head displacement during passive mobility testing in pronation, supination, and neutral rotation using an electromagnetic tracking device. Measurements at various ligament sectioning stages and ulnar angulation substages were statistically compared with those in the intact elbow.

Radial head displacement increased with sequential ligament sectioning and increased proportionally with the degree of anterior ulnar angulation. Annular ligament sectioning resulted in a significant increase in displacement only in pronation (*P* < .05). When the anterior ulnar deformity was reproduced, the radial head displaced least in supination. The addition of proximal interosseous membrane sectioning significantly increased the radial head displacement in supination (*P* < .05), regardless of the degree of anterior ulnar angulation.

Our Monteggia fracture model showed that radial head instability is influenced by the degree of soft tissue damage and ulnar angulation. Annular ligament injury combined with a minimal (5°) ulnar deformity may cause elbow instability, especially in pronation. The proximal interosseous membrane contributes to radial head stability in supination, regardless of ulnar angulation, and proximal interosseous membrane injury led to significant radial head instability in supination.

## 1 Introduction

A Monteggia fracture is a common forearm injury comprising an ulnar fracture with radial head dislocation. Bado classified Monteggia fractures into four types according to the direction of radial head dislocation and ulnar shaft angulation.^[1]^ The Bado type I fracture (anterior radial head dislocation and ulnar angulation) is the most common type (60%–70%).^[2]^

Monteggia fractures are sometimes missed,^[3–5]^ and chronic lesions are difficult to treat.^[6–8]^ One study found that interosseous membrane (IOM) integrity is essential for radial head stabilization when there is recurrent dislocation.^[9]^ Other reports indicate that the force transmitted through the fractured ulna might result in rupture of the annular ligament and proximal portion of the IOM with subsequent acute radial head dislocation.^[10]^ Further, the tautness pattern of the IOM varies according to the Bado classification.^[6]^ Thus, when managing a Monteggia fracture, various soft tissue injuries, including proximal IOM damage, must be considered. Although many clinical^[11–14]^ and biomechanical studies^[15–21]^ have described Monteggia fractures, few have examined the correlation between the degree of soft tissue damage and radial head stability^[9]^; moreover, the influence of the magnitude of ulnar angulation on elbow instability is unknown.

A clearer understanding of the relationship between radial head instability and soft tissue damage and ulnar angulation deformity could facilitate surgical treatment planning in patients with forearm fractures. We hypothesized that radial head instability correlates with the magnitude of ulnar angular deformity and the degree of proximal forearm soft tissue injury in Bado type I Monteggia fractures. We investigated this hypothesis by testing the radial head instability by reproducing ulnar angular deformity and proximal forearm soft tissue injury on cadaveric upper extremities.

## 2 Materials and methods

### 2.1 Specimen preparation

The Chiang Mai University Faculty of Medicine Research Ethics Committee approved this study on human cadavers (code: ORT-2558-03512). The study was performed in accordance with the Ethical Standards of the 1964 Declaration of Helsinki.

We improved and adapted the methods we developed in our previously published biomechanical study of radial head dislocation^[22]^ for the Bado type I Monteggia fracture model used in this study. We used 6 fresh-frozen cadaveric upper extremities (5 males; mean decedent age, 66 years [range, 51–79]). Gross examination of the specimens revealed no skeletal or articular pathology of the upper extremity. All limbs were thawed at room temperature (mean, 22°C) before use and kept moist with normal saline spray during the experiment. Specimens were amputated above the elbow, and the wrists were then disarticulated. We preserved the ligaments, joint capsules (stabilizing the distal and proximal radioulnar joints), IOM, and distal biceps tendon. All other soft tissue was removed.

### 2.2 Experimental setup

**Figure 1:**
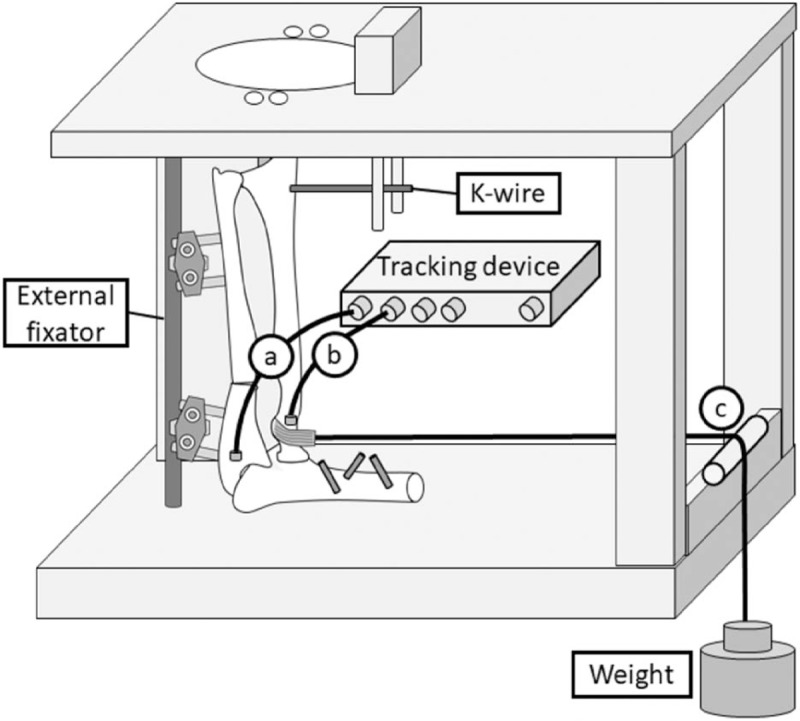
Bado type I fracture model diagram. The ulna is secured with an external fixator, and the humerus is fixed on the jig. A 2-mm K-wire is inserted in the distal radius to hold the limb in each forearm rotation (supination, neutral rotation, and pronation). (A) Proximal ulnar sensor, (B) radial head sensor, and (C) pulley system (sling, pulley, and weight).

The humerus was firmly fixed on a customized wooden jig, and the ulna was fixed with an external fixator, with the elbow flexed at 90°. The radius was held in each forearm position (full pronation, neutral rotation, and full supination) with a 2-mm K-wire inserted into the distal radius (Fig. 1). The bar of the customized jig held the K-wire in the sagittal plane only, and the horizontal movement of the radial head was not impaired. Neutral rotation was determined as the mid-point of full pronation and supination. We measured radial head displacement relative to the proximal ulna with an electromagnetic tracking device (trakSTAR; Ascension Technology Corporation, Shelburne, VT). The sensors were placed in short, 3-mm-diameter polycarbonate tubes to stabilize and protect them after the tubes were inserted into bone holes made using a 3.5-mm-diameter drill. The radial head sensor was advanced from the proximal metaphysis to the radial head to avoid interference from radial head rotation. We reproduced force applied to the radial head using a system consisting of a sling, pulley, and weight to pull the biceps tendon parallel to the long axis of the humerus with a force of 20 N.

### 2.3 Ulnar deformity and ligament injury model

**Figure 2:**
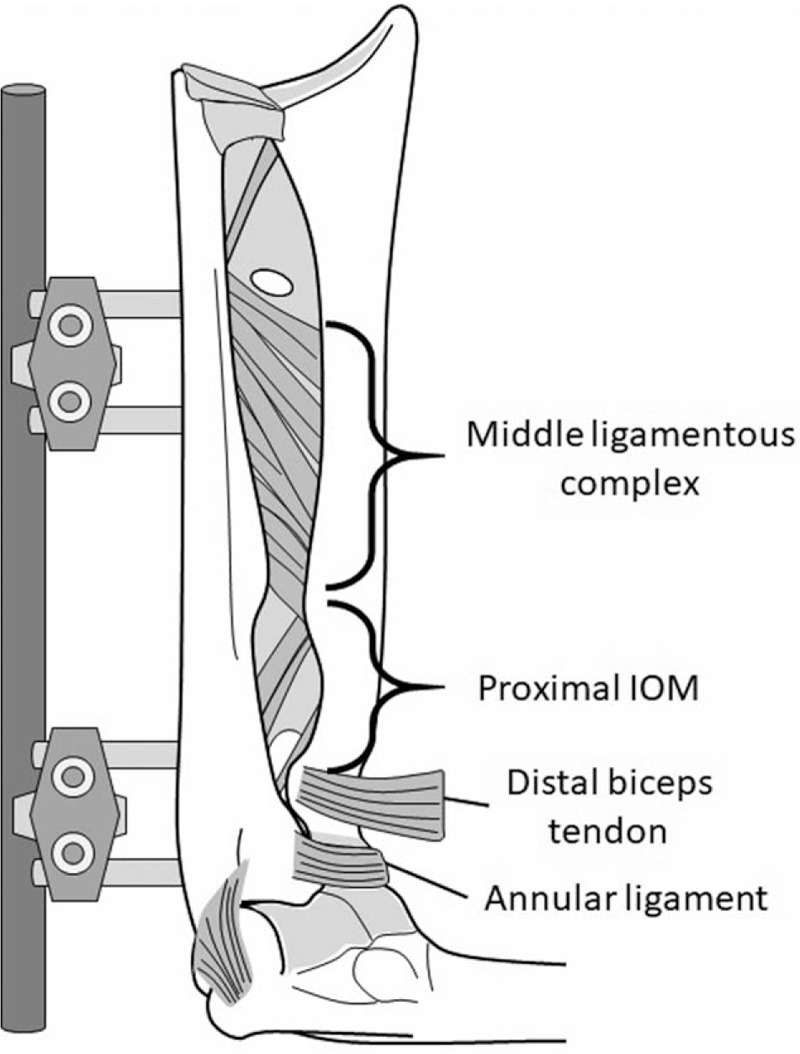
Monteggia fracture simulation. Stage 0: the elbow shows capsular sectioning and the ligaments are intact. IOM = interosseous membrane.

**Figure 3:**
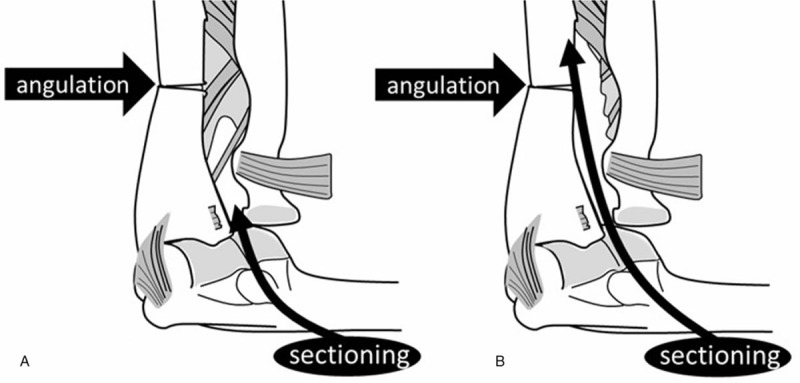
Monteggia fracture simulation. (A) Stage 1: the annular ligament is completely sectioned. (B) Stage 2: the membrane and cords are divided. The point of proximal ulnar angulation is identified in both images (arrows).

We performed sequential sectioning of the annular ligament and proximal IOM to simulate radial head instability. The proximal IOM is defined as the proximal membranous portion on the proximal side of the middle ligamentous complex of the IOM, and it contains the proximal oblique and dorsal oblique accessory cords.^[23]^ Elbow ligament and IOM sectioning stages were defined as follows: stage 0, intact elbow with capsular sectioning (Fig. 2); stage 1, elbow with annular ligament sectioned (Fig. 3A); and stage 2, elbow with proximal IOM sectioned (Fig. 3B). In stage 1, the annular ligament was sectioned completely with preservation of the lateral ulnar collateral ligament. In stage 2, the membrane and cords were divided from the radial head neck to the proximal side of the middle ligamentous complex. In stages 1 and 2, we simulated an angulation deformity of the proximal one-third of the ulna, proximal to the proximal end of the ulnar origin of the middle ligamentous complex in all specimens (Fig. 2) from 0° to 20° with the following 5° substages: substage a, 0°; substage b, 5°; substage c, 10°; substage d, 15°; and substage e, 20°. These anterior angulation deformities were produced by ulnar osteotomy and external fixation using a goniometer. The same surgeon reproduced this model in all specimens.

### 2.4 Passive mobility testing and data acquisition

We tested passive mobility by pulling the distal biceps tendon anteriorly because anterior radial head dislocation is clinically associated with the force applied by the biceps tendon.^[24–27]^ Radial head displacement was defined as the distance from the preload position before ligament sectioning and ulnar deformation to the post-load position after simulating ligament and ulnar injuries because extensive ulnar deformity and ligament sectioning resulted in radial head displacement, even before loading. The radial head displacement ratio was calculated as the percentage of radial head displacement relative to the radial head diameter. We measured the location of the radial head in full pronation, neutral rotation, and full supination before and after loading. Each measurement was repeated at each sectioning stage and ulnar angulation substage.

### 2.5 Statistical analysis

We reported the measurements as mean ± standard deviation. SPSS for Windows version 22.0 (IBM Corp, Armonk, NY) was used for the statistical analyses. Two-way repeated-measures analysis of variance was used to assess the radial head displacement ratio. We then performed the Dunnett multiple comparison test to compare stage 1 and 2 substage data with stage 0 results. Statistical differences between stages 1 and 2 in each substage were analyzed by a 2-sided paired *t* test. We considered a *P* value < .05 statistically significant.

## 3 Results

**Figure 4:**
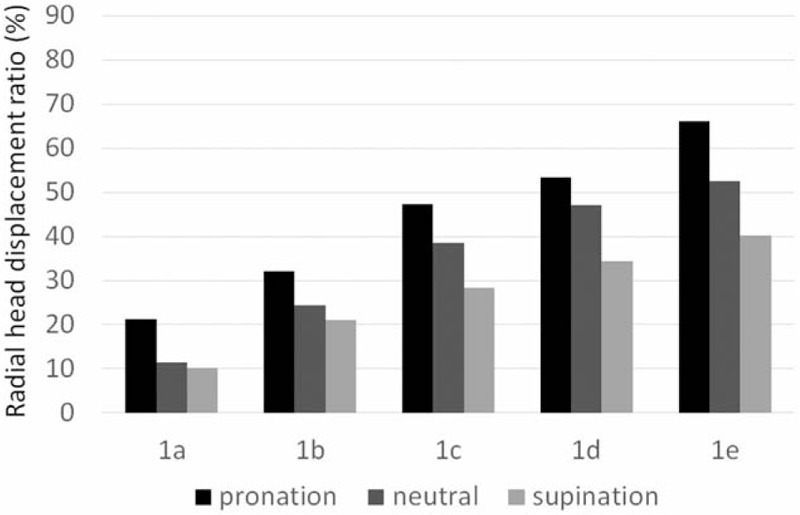
Radial head displacement ratio in the elbow after annular ligament sectioning. In each angulation deformity substage, the radial head displacement ratio is the greatest in pronation and least in supination (1a, 0°; 1b, 5°; 1c, 10°; 1d, 15°; 1e, 20°).

Stage 0 sectioning resulted in a displacement ratio of <5% in all forearm positions. After stage 1 sectioning, the displacement ratio increased proportionally according to the degree of anterior ulnar angulation and was greater in pronation than in neutral rotation or supination (Fig. 4). In neutral rotation, there was no significant radial head displacement after stage 1a sectioning (11% ± 5%, *P* > .05; Table 1), whereas the displacement ratio was significantly greater in stage 1b (24% ± 10%, *P *< .05) than in stage 0. In pronation, the displacement ratio was significantly greater in stage 1a (21% ± 6%, *P *< .05) than in stage 0 (Table 2). In supination, the displacement ratio was significantly greater in stage 1c (28% ± 18%, *P* < .05) than in stage 0 (Table 3).

**Table 1:**
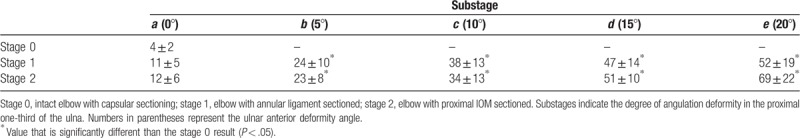
Radial head displacement ratio in neutral rotation in a Bado type I Monteggia fracture model.

**Table 2:**
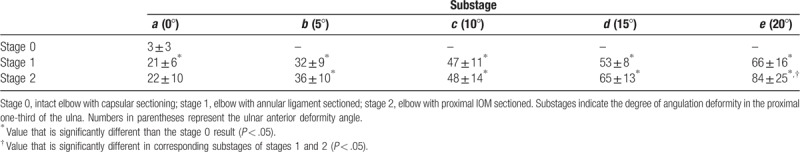
Radial head displacement ratio in pronation in a Bado type I Monteggia fracture model.

**Table 3:**
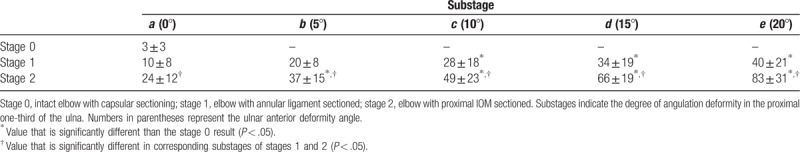
Radial head displacement ratio in supination in a Bado type I Monteggia fracture model.

**Figure 5:**
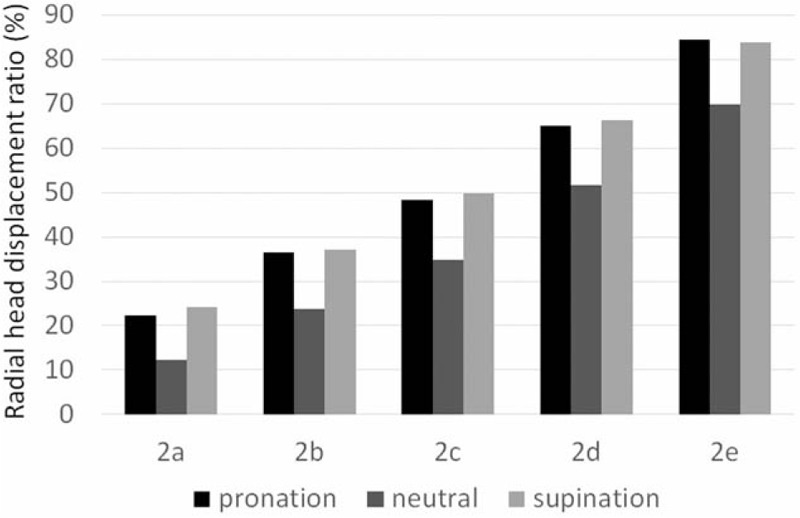
Radial head displacement ratio in the elbow after proximal interosseous membrane sectioning. In each substage, the radial head displacement ratio is the least in neutral rotation (2a, 0°; 2b, 5°; 2c, 10°; 2d, 15°; 2e, 20°).

After stage 2 sectioning, there was a proportional relationship between the displacement ratio and the degree of anterior ulnar angulation, similar to that found in stage 1. Further, our comparison of stage 2 substage results in the 3 positions showed that the radial head displacement ratio was the lowest when the forearm was in neutral rotation, regardless of the substage (Fig. 5). Our substage comparison of radial head displacement in supination showed significantly greater displacement in stage 2 than in stage 1 in all substages (Table 3).

In neutral rotation, the displacement ratio was significantly greater in stages 2b (24% ± 8%, *P* < .05) and 2c-e (*P* < .05) than in stage 0 (Table 1); however, there were no significant differences between the corresponding substages of stages 1 and 2. In pronated limbs, the displacement ratio was significantly greater in stages 2b (36% ± 10%, *P* < .05) and 2c-e (*P* < .05) than in stage 0 (Table 2), and the displacement ratio was significantly different between stages 1e and 2e (*P* < .05). In supinated limbs, the displacement ratio was significantly greater in stages 2b (37% ± 15%, *P* < .05) and 2c-e (*P* < .05) than in stage 0 (Table 3). There were significant differences in the displacement ratios of all stage 1 and 2 substages during supination.

## 4 Discussion

In this biomechanical study, we found that radial head instability correlated with the magnitude of ulnar angular head deformity and the degree of soft tissue injury in the proximal forearm in a cadaveric Monteggia fracture model. The annular ligament is a primary stabilizer of the radial head.^[28]^ In the current experiment, specimens without an ulnar angular deformity showed no significant change in the radial head displacement ratio in stages 1 and 2, except in supination. These findings suggest that the middle ligamentous complex (central band) of the IOM connecting the radius and ulna prevents anterior radial head instability if there is no bony injury. This conclusion is supported by a clinical case series that showed that annular ligament repair is not essential in the operative treatment for radial head injuries.^[29]^ When the magnitude of anterior ulnar angulation increased in association with ligament sectioning, the radial head displacement increased proportionally. The mechanism of this increased instability can be explained by the subsequent IOM dysfunction associated with ulnar deformity. Because anterior angulation of the ulna shortens the radioulnar distance, subsequent IOM loosening and dysfunction may lead to radial head instability.

Sandman et al^[18]^ evaluated the radial head displacement associated with an ulnar deformity in a biomechanical study and showed that displacement increased as ulnar malalignment progressed. However, they only used lateral radiography to measure displacement and did not evaluate differences related to forearm position. In the present study, we measured 3-dimensional radial head displacement with an electromagnetic tracking device. We found that the magnitude of radial head instability increased proportionally and significantly correlated with the magnitude of ulnar deformity. Moreover, we identified a different instability pattern in each forearm position. The significant radial head instability that occurred with minimal ulnar deformity (5°) indicates that accurate anatomical reduction of the ulnar fracture is required to prevent radial head instability in patients with an acute Monteggia fracture.

There is no consensus regarding forearm position during the immobilization period after radial head reduction.^[12,13,24,30]^ Of the 3 positions we evaluated in our Monteggia fracture model, in the absence of ulnar angulation, only pronation was associated with significant radial head instability after annular ligament sectioning. This result indicates that physicians should avoid immobilizing the forearm in pronation after the radial head and ulnar deformities are reduced in patients with a Bado type I Monteggia fracture. After the addition of proximal IOM sectioning, there was a significant increase in radial head instability in supination and a slight increase in neutral rotation. These findings indicate that soft tissue injury may cause positional radial head instability. This information could be useful in diagnosing the pattern of soft tissue injury and planning treatment. In a clinical setting, the degree of soft tissue damage associated with a Monteggia fracture may be determined based on the positions in which the forearm is stable after the radial head dislocation is reduced. If the proximal IOM injury is involved, the residual radial head instability would remain, regardless of forearm rotation; therefore, an additional surgical procedure may be required along with the reduction of ulnar deformity.

The central band of the middle ligamentous complex has the strongest fibers connecting the radius and ulna.^[31]^ Additionally, the strain on the central band and pressure on the proximal radioulnar joint are the greatest in the neutral rotation position.^[16,17]^ Although the distal oblique bundle of the distal IOM is known to contribute to distal radioulnar joint stability,^[32,33]^ the role of the proximal IOM in proximal radioulnar joint stability is not well described. In the present study, there was a significant difference between all substages of stages 1 and 2 in this position, indicating that the proximal IOM contributes to anterior radial head stability in supination, regardless of the degree of ulnar deformity. The phenomenon that the anterior radial head instability changes along the forearm rotation is clinically well known in case of Monteggia fracture, and we proved that it is related to the proximal IOM.

This study had several limitations. First, although we reproduced a Bado type I fracture by pulling the distal biceps tendon in the anterior direction, this model cannot reproduce other types of fracture-dislocations because a posterolateral radial head dislocation does not result from a pull from the biceps tendon. Second, we did not assess the dynamic effects of the muscles around the forearm bones. Both supinator and pronator muscles are dynamic radial head stabilizers, and in vivo displacement of the radial head may be less significant than the displacement we observed. Third, repeatedly overloading the soft tissue may have caused it to become increasingly loose as the experiment progressed; we applied a load of 20 N to the biceps tendon (the load amount used in previous biomechanical studies) to minimize this change.^[21,23,34,35]^ Fourth, our study was limited by its small sample size, which is the usual limitation of biomechanical studies. We used 6 cadavers, which was the maximum sample size that our study schedule would allow. Despite this limitation, we proved that, in various stages and substages, the radial head significantly displaced from stage 0. We believe that we could show the clinical significance of radial head instability with the present study. Finally, we did not consider the incidence of bone diseases (such as osteoporosis) in our cadavers. We believed that the radial head instability was mostly affected by the ulnar deformity and soft tissue injury. The fracture pattern of the ulna was clearly affected by the bone disease; however, our study was not aimed at investigating the pathology of the ulnar fracture pattern. Further studies should consider and improve these limitations to strengthen the results’ implications.

In conclusion, the cadaveric Bado type I Monteggia fracture model used in this study revealed that radial head instability correlated with the degree of ulnar angular deformity and the extent of soft tissue sectioning. Significant anterior radial head instability occurred after sectioning the annular ligament in limbs with a moderate ulnar deformity (>5° anterior angulation) due to IOM loosening. Therefore, surgeons should reduce ulnar deformities anatomically. The proximal IOM contributes to radial head stability in supination, regardless of ulnar angulation, and proximal IOM injury led to significant radial head instability in supination. This information will be valuable in diagnosing the pattern of soft tissue injury and planning detailed treatment.

## Acknowledgments

The authors thank the staff of the Excellence in Osteology Research and Training Center (ORTC) Surgical Training Center, the Department of Anatomy, and the Department of Orthopaedics of Chiang Mai University for their assistance in this study.

## Author contributions

**Conceptualization:** Naoki Hayami, Shohei Omokawa.

**Data curation:** Naoki Hayami, Akio Iida, Tsutomu Kira.

**Formal analysis:** Naoki Hayami, Akio Iida, Tsutomu Kira.

**Funding acquisition:** Naoki Hayami.

**Investigation:** Naoki Hayami, Pasuk Mahakkanukrauh, Jirachart Kraisarin.

**Methodology:** Hisao Moritomo, Takamasa Shimizu, Kenji Kawamura.

**Project administration:** Shohei Omokawa, Pasuk Mahakkanukrauh.

**Resources:** Pasuk Mahakkanukrauh, Jirachart Kraisarin.

**Software:** Naoki Hayami, Shohei Omokawa.

**Supervision:** Shohei Omokawa, Hisao Moritomo, Pasuk Mahakkanukrauh, Yasuhito Tanaka.

**Validation:** Shohei Omokawa, Hisao Moritomo, Pasuk Mahakkanukrauh, Yasuhito Tanaka.

**Visualization:** Naoki Hayami.

**Writing – original draft:** Naoki Hayami, Takamasa Shimizu, Kenji Kawamura.

**Writing – review and editing:** Naoki Hayami, Shohei Omokawa, Kenji Kawamura, Yasuhito Tanaka.
